# Feasibility and effects of adapted cardiac rehabilitation after stroke: a prospective trial

**DOI:** 10.1186/1471-2377-10-40

**Published:** 2010-06-09

**Authors:** Ada Tang, Susan Marzolini, Paul Oh, William E McIlroy, Dina Brooks

**Affiliations:** 1 Department of Physical Therapy, University of Toronto, Toronto, Canada; 2 Institute of Medical Science, University of Toronto, Toronto, Canada; 3 Toronto Rehabilitation Institute, Toronto, Canada; 4 Heart and Stroke Foundation of Ontario Centre for Stroke Recovery, Sunnybrook Health Sciences Centre, Toronto, Canada; 5 Department of Kinesiology, Faculty of Applied Health Sciences, University of Waterloo, Waterloo, Canada

## Abstract

**Background:**

Despite the cardiovascular etiology of stroke, exercise and risk factor modification programs akin to cardiac rehabilitation (CR) are not available. This study aimed to establish the feasibility of adapting a CR model for individuals with mild to moderate stroke disability. A secondary objective was to determine the program's effects on aerobic and walking capacity, and stroke risk factors.

**Methods:**

A repeated measures design was used with a 3-month baseline period and 6-month adapted CR intervention (n = 43, mean ± SD age 65 ± 12 years, 30 ± 28 months post stroke). Feasibility was determined by the number of participants who completed the study, occurrence of adverse events and frequency, duration and intensity of exercise performed. To determine effectiveness of the program, outcomes measured included aerobic capacity (VO_2_peak, ventilatory threshold), 6-Minute Walk Test (6MWT) distance, and risk factors. Descriptive statistics characterized the classes attended and number and intensity of exercise sessions. Paired *t*-tests, one-factor repeated measures analyses of variance contrasts and chi-square analyses were used to compare changes over time.

**Results:**

Two participants withdrew during the baseline period. Of the remaining 41 participants who commenced the program, 38 (93%) completed all aspects. No serious adverse effects occurred. Post-intervention, VO_2_peak improved relative to the stable baseline period (*P *= 0.046) and the increase in ventilatory threshold approached significance (*P *= 0.062).

**Conclusions:**

CR is feasible after stroke and may be adapted to accommodate for those with a range of post-stroke disability. It is effective in increasing aerobic capacity. CR may be an untapped opportunity for stroke survivors to access programs of exercise and risk factor modification to lower future event risk.

**Trial registration:**

ClinicalTrials.gov registration number: NCT01067495

## Background

Stroke is the leading cause of neurological disability in adults [1]. Poor levels of fitness, including low aerobic capacity [2] can compound the challenges by further limiting the ability to engage in many daily activities and impacting risk of subsequent stroke. Traditional stroke rehabilitation is effective in improving functional independence [3] yet insufficiently challenges the cardiorespiratory system to induce aerobic benefit [4].

In contrast, cardiac rehabilitation (CR) is focused on exercise and risk factor management and is part of routine care for individuals with cardiac disease. Similar programs are lacking for the stroke population. Parallels between stroke and heart disease in cardiovascular etiology, co-morbidities and risk factors suggest that a CR model of care may provide stroke survivors much-needed health promotion opportunities for secondary prevention.

A key challenge remains in the adaptability of traditional CR to accommodate participants with moderate levels of disability from stroke. Neurological deficits commonly impact on the ability to engage in exercise such as walking, and alternative modes of aerobic training (e.g. stationary cycling) must be considered. Appropriate staff training and conducting classes with lower staff-to-participant ratios [5] may accommodate for stroke-related issues such as cognitive or communication impairment, issues not typically encountered in traditional CR.

Therefore, the primary objective was to establish the feasibility of an adapted CR model of care with stroke survivors with mild to moderate impairment. The secondary objective was to determine the effects of this rehabilitation model on aerobic and walking capacity, and stroke risk factors.

## Methods

**Figure 1:**
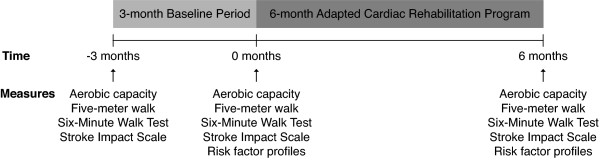
**Study design**. Schematic of time points for data collection and overall study design

The study was conducted as part of an outpatient CR program in a rehabilitation facility. Study procedures were approved by local university and hospital research ethics committees. Informed written consent was obtained from all participants. A repeated-measures design was used with a 3-month monitoring period without intervention, followed by a 6-month adapted CR program (Figure 1).

### Participants

Individuals at least 3 months post-stroke were recruited from stroke rehabilitation programs, outpatient follow-up clinics and from the community, and were eligible if they were able to provide informed consent, understand the evaluation procedures, walk more than 10 meters independently with or without aids and have a Chedoke-McMaster Stroke Assessment [6] leg impairment score of 3 (marked spasticity and weakness) or higher (representing less impairment) at the start of the program. Exclusion criteria included contraindications to maximal exercise testing [7], or significant musculoskeletal, pain, cognitive or behavioural issues that would limit the ability to perform the exercise testing and/or participate in the program.

### Intervention

The adapted CR, conducted at the Toronto Rehabilitation Institute's Cardiac Rehabilitation and Secondary Prevention Program, was modeled after its traditional program [8]: once-weekly 90-minute sessions of education and supervised, individually-prescribed aerobic and resistance exercise training were supplemented by 4 additional aerobic and 1-2 additional resistance training sessions conducted at home. All training sessions were tracked via weekly exercise diaries. The 1:5 staff-to-participant ratio used in the adapted model was lower than the usual 1:12 ratio, and class size of 20 participants was lower than the typical 80-100 [8]. These adaptations permitted adequate supervision yet facilitated progressively greater independence over time.

*Aerobic exercise *was prescribed 5 days per week. Initial training intensity was established based on a combination of first calculating 60-80% of heart rate reserve (HRR) [9], which was then compared to the heart rate at the ventilatory anaerobic threshold (VAT) [10] to ensure agreement between these methods. Finally, the intensity of exercise may have been adjusted to achieve an 11-14 rating of perceived exertion (RPE) [11]. For this adapted program, heart rate, rather than VO_2_, was used to guide training intensity as oxygen requirements for various activities are significantly higher for those with physical impairments compared to non-impaired individuals; individuals post-stroke may need to expend up to 2 times more energy than people without stroke walking at the same pace [12].

Aerobic exercise progressed up to 30-60 minutes per session. Exercise intensity was monitored using the final HR (via 10-second pulse rate taken at the radial artery or HR monitor) and RPE taken at the end of the training session. The weekly average training HR was calculated from the final HR values recorded on the exercise diaries.

Training modality was individually prescribed. Over ground walking was preferred and was typical of the traditional CR program. Walking was prescribed if ambulation capacity was at sufficiently high speeds and duration to accrue aerobic benefit. The program was adapted to use upright and semi-recumbent cycle ergometers when stroke-related balance deficits and lower limb dys-control precluded walking programs [13]. Other adaptations included the use of interval training (short bouts of higher exercise intensity performed between longer periods of lower intensity exercise) for those unable to sustain faster walking or cycling pace for prolonged periods (n = 5), or dividing exercise across 2 sessions within the same day if initial tolerance was low. On the exercise diaries, total exercise time and final training HR were recorded, regardless of the method of training.

*Resistance training *was prescribed 2-3 sessions per week for 7-10 upper and lower body exercises. Initial weight loads of 60% of 1-repetition maximum were used for the non-paretic limb. For the paretic limb, a necessary adaptation was use of the rating of perceived exertion (11-14) rather than 1-repetition maximum to guide intensity. One set of 10 repetitions was prescribed initially, and participants progressed to 2 sets and increased to 15 repetitions before weights increased. Resistance was provided through hand held dumbbells, resistance bands, body weight or weight machines. Assessment of functional abilities, degree of hypertonicity, available range of motion and severity of balance impairment were considered.

*Education sessions *were held 1-2 times monthly and included many topics standard to the traditional CR stream (heart-healthy diet, diabetes management, or blood pressure (BP) control). Other sessions were specifically designed or adapted for the stroke participants, including sleep apnea and stroke risk or considerations for purchasing home exercise equipment after stroke.

### Measures

#### A. Feasibility

Feasibility was determined by the number of participants who completed the study, occurrence of adverse events and frequency, duration and intensity of exercise as recorded on weekly diaries. As a conservative estimate, if a diary was not submitted, it was assumed that no exercise was performed that week.

At the end of the study, participants' satisfaction with the program was evaluated by asking, on a scale of 1 (Poor) to 5 (Excellent), "Please rate the following components of the program: 1) aerobic exercise sessions, 2) strength exercise sessions and 3) education sessions".

Other outcome measures were completed at 3 possible time points: 1) start of the baseline period (Pre-Baseline, -3 months), 2) end of the baseline and start of intervention (Pre-Program, 0 months) and 3) end of the intervention (Post-Program, 6 months) (Figure 1).

#### B. Aerobic capacity

A symptom-limited graded maximal exercise test determined peak oxygen uptake (VO_2_peak), peak work rate and VAT. Resting HR and BP were also noted.

Ten (23%) of the 43 participants were deemed 'higher risk' because of cardiac history or presence of cardiovascular symptoms (e.g. myocardial infarction, angina, dysrhythmias), thus were tested at the CR cardiopulmonary assessment laboratory [8]. Of these 10 participants, 6 were tested using an upright cycle ergometer, 3 with a recumbent cycle ergometer and 1 using a modified Bruce treadmill protocol [14]. The 33 (77%) remaining participants were tested in the stroke research laboratory using a semi-recumbent cycle ergometer. The protocol was based on one used previously [13], modified to use 5-, 10- or 15-watt incremental increases in work rate to maintain total test time between 8-10 minutes. VAT, defined as the point where ventilation increases at a greater rate than oxygen uptake [15], was determined graphically [10] using 2 independent assessors.

#### C. Six-minute walk test (6MWT)

Standardized instructions [16] were given to walk as far as possible over a 30-meter course in 6 minutes. Gait aids were permitted. A practice trial was performed before the actual test [17]. The distance covered was the primary outcome. Distance was also expressed as a percentage of predicted values based on age, sex, weight and height [18].

#### D. Cardiovascular disease risk factor profiles

Resting BP was measured at all 3 time points as part of the maximal exercise test. Measurement of waist and hip circumference, percent body fat by bioelectrical impedance analysis, fasting blood glucose, glycated haemoglobin, total, high- (HDL) and low-density lipoprotein (LDL) cholesterol and triglyceride levels were made at pre- and post-intervention only as these assessments were part of routine practice in the traditional CR program. Participants who met the criteria for the metabolic syndrome [19] were identified.

### Analysis

A sample size of 27 was chosen based on calculations to detect a clinically important difference of 54 meters in the 6MWT among individuals with chronic obstructive pulmonary disease [20] (two-tailed type I error 0.05, type II error 90%, SD 96). To account for attrition and to provide an opportunity to detect changes in other primary outcomes, we aimed to recruit over 30 participants.

Descriptive statistics were performed on participants' baseline characteristics, classes attended, number and intensity of exercise sessions completed, and on all measures at each available time point. To determine differences over time for continuous variables, paired *t*-tests (change from 0 to 6 months) or one-factor repeated measures analyses of variance contrasts (change over baseline period (-3 to 0 months), and from baseline (-3, 0 months) to the end of the program (6 months)) were used. Chi-square analyses compared changes over time for non-continuous variables. Statistical Analysis Software Version 9.1 was used with a significance level of *P *< 0.05.

## Results

### Study sample

**Figure 2:**
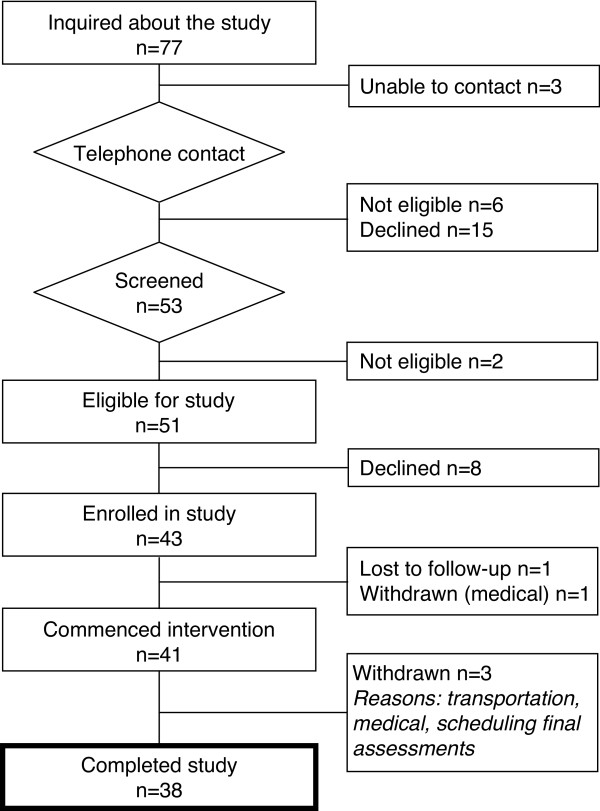
**Participant flow chart**. Flow of participants through the study

Figure 2 depicts the flow of recruitment, enrolment and study completion. Forty-three participants enrolled (Table 1). After the first test (Pre-Baseline, -3 months), 2 participants were removed from further analysis (medical issues unrelated to study, loss to follow up) (Figure 2). The remaining 41 participants demonstrated no changes in the primary outcome measures over the baseline period (*P *= 0.09-0.98), and all commenced the adapted CR program.

**Table 1:** Participant characteristics

Variable	n (%) or mean ± SD (range)
Men	30 (70%)
Age, years	64.5 ± 12.2 (38-86)
Time post-stroke, months	30 ± 27.3 (3-113)
Ischemic/Hemorrhagic/Unknown stroke type	27 (63%)/11 (26%)/5 (12%)
Right/Left/Bilateral hemisphere affected	17 (40%)/24 (56%)/2 (5%)
Cardiovascular history	
Prior stroke/transient ischemic attack	5 (12%)/2 (5%)
Atrial fibrillation	7 (16%)
Myocardial infarction	1 (2%)
Left ventricular hypertrophy	1 (2%)
Coronary artery bypass graft surgery	4 (9%)
Percutaneous coronary intervention	1 (2%)
Diabetes	12 (28%)
Hypertension	28 (65%)
Dyslipidemia	34 (79%)
Never smoked/Remote smoker/Currently smoking	29 (67%)/9 (21%)/5 (12%)
Gait aids, None/Cane/Walker/Rollator	17 (40%)/18 (42%)/1 (2%)/7 (16%)
National Institutes of Health stroke scale score	2.9 ± 2.7 (0-11)
Chedoke-McMaster Stroke Assessment scores	
Arm/Hand	4.4 ± 1.6 (2-7)/4.6 ± 1.8 (2-7)
Leg/Foot	5.1 ± 1.3 (2-7)/4.4 ± 1.7 (2-7)
Berg Balance Scale score	46.4 ± 11.6 (4-56)
5-Meter Walk	
Self-paced gait speed, cm/s	76.8 ± 35.5 (20.4-152.3)
Fast-paced gait speed, cm/s	85.2 ± 37.4 (28.3-169.7)
Calculated gait symmetry ratio, self-paced walk	1.50 ± 0.6 (1.01-3.85)
Calculated gait symmetry ratio, fast-paced walk	1.49 ± 0.49 (1.02-2.63)

Baseline characteristics for all participants, n = 43

While participants' stroke severity was primarily mild according to the National Institutes of Health stroke scale [21] (29 (71%) mild, 12 (29%) moderate, 0 severe), mobility-related outcomes described otherwise. Participants walked 56% of predicted 6MWT distance [18], 17 (42%) were classified as household or limited community ambulators based on gait speed [22], and 11 (27%) presented with severe gait asymmetry [23].

### Feasibility of adapted CR after stroke

Of the 41 people who started the adapted CR program, 38 (93%) completed the study (Figure 2). Three participants withdrew due to the following: transportation issue; medical issue unrelated to study; inability to schedule final study assessments.

Two medical events occurred during the course of the study that temporarily affected program participation: 1) flare-up of chronic hip bursitis managed with stretching and adapting exercises to minimize pain and inflammation, and 2) onset of low-back and hip pain managed with postural education and modifying aerobic training to stationary cycling instead of walking. Both issues resolved and the exercise programs were completed without interruption.

Aerobic exercise prescriptions progressed from an initial 20.1 ± 6.9 minutes of exercise per session to 28 ± 10.2 minutes by the end of the program (*P *< 0.0001). Walking was prescribed for 26 people (over ground walking n = 25, treadmill n = 1), 11 used cycle ergometry and 1 person had a combination program of walking and cycling. Participants in the cycle ergometry group had lower Chedoke-McMaster Stroke Assessment leg scores compared to the other groups (4.4 ± 1.3 vs. 5.5 ± 1.3, *P *= 0.03).

Class attendance was 83.5 ± 11.5%, with 31 (82%) participants attending ≥75% of classes. According to the 76.6 ± 19.6% of diaries submitted, participants exercised a median of 4 (range 0-7) aerobic sessions (76 ± 52 minutes) per week, at an intensity of 48.9 ± 23.2% (range 0-92%) of HRR. This represented 63 ± 28.2% of exercise time prescribed.

In response to the post-program satisfaction survey (n = 34), median (range) ratings for the aerobic training, resistance training and education sessions were 5 (1-5), 5 (1-5) and 5 (2-5) respectively.

### Effect of adapted CR on aerobic and walking capacity, and risk factors

**Figure 3:**
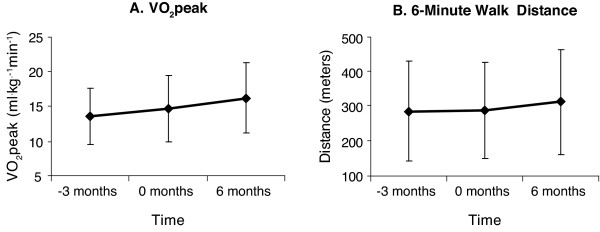
**Changes in aerobic capacity and walking function**. Changes in A) VO_2_peak and B) 6-minute walk distance pre-baseline (-3 months), and pre- and post-program (0, 6 months). Error bars indicate standard deviation.

VO_2_peak improved significantly at end of the CR program relative to the baseline period (*P *= 0.046) and the increase in VAT approached significance (*P *= 0.062) (Table 2, Figure 3). Post hoc analyses comparing change scores in VO_2_peak and VAT among participant groups who trained aerobically through walking versus cycling did not reveal any significant results (VO_2_peak change among cyclers (n = 11) vs. walkers (n = 27, including the participant who trained via walking and cycling) 1.5 and 1.4 ml·kg^-1^·min^-1 ^respectively (*P *= 0.91), VAT change 1.8 and 1.5 ml·kg^-1^·min^-1 ^respectively (*P *= 0.96)).

**Table 2:** Changes in study outcomes measured across 3 time points

					*P *values	
					
	n	Pre-Baseline -3 months	Pre-Program 0 months	Post-Program 6 months	ANOVA contrast for Pre-Baseline (-3 months) vs. Pre-Program (0 months)	ANOVA contrast for Baseline Period (-3, 0 months) vs. Post-Program (6 months)
**Aerobic capacity**						
VO_2_peak, ml·kg^-1^·min^-1^	32	13.6 ± 4.1 (5.8-22.7)	14.8 ± 4.8 (7-24.5)	16.2 ± 5.1 (7.4-27.9)	0.301	0.046
Peak workload, watts	36	60.4 ± 27.8 (15-120)	63.1 ± 31.2 (20-130)	64.6 ± 33.2 (20-130)	0.709	0.647
Ventilatory anaerobic threshold, ml·kg^-1^·min^-1^	27	10.3 ± 2.7 (4.8-14.9)	11 ± 3.2 (5.9-19.5)	11.9 ± 3.0 (6.1-18.5)	0.335	0.062
Resting heart rate, beats/minute	38	73.9 ± 13.8 (50-106)	73.4 ± 12.5 (52-103)	71.6 ± 14.8 (44-102)	0.877	0.459
Resting blood pressure						
Systolic, mmHg	38	121 ± 13.7 (100-170)	122.6 ± 15.7 (89-158)	122.8 ± 17.3 (93-184)	0.66	0.74
Diastolic, mmHg	38	76.4 ± 11.4 (54-94)	74.8 ± 10.9 (52-98)	77.3 ± 10 (41-100)	0.51	0.431
**Walking capacity**						
6MWT distance, meters	37	284.2 ± 145.8 (30-547)	286.4 ± 140 (45-576)	311 ± 152.1 (55-585)	0.948	0.382

Changes in study outcomes across 3 time points: pre-baseline (-3 months), and pre- and post-program (0, 6 months)

Values are mean ± SD (range). Abbreviations: 6MWT = 6-Minute Walk Test; SIS = Stroke Impact Scale.

While statistically non-significant, average change in 6MWT distance from -3 to 0 months was 2.2 meters, and the change from 0 to 6 months was 24.6 meters (Table 2, Figure 3). Secondary subgroup analysis conducted to determine group differences based on training modality did not reveal any significant results (change 21 vs. 24 meters for cyclers and walkers respectively, *P *= 0.20).

There were no post-program changes in risk factors (Table 3), although slight reductions in percent body fat (change 0.3%) and fasting plasma glucose (change 0.24 mmol/L) were observed. Two participants who initially met the criteria for the metabolic syndrome no longer did so by the end of the study.

**Table 3:** Changes in study outcomes measured at 2 time points

	n	Pre-Program 0 months	Post-Program 6 months	*P *values for Pre-Baseline (0 months) vs. Post-Program (6 months)
**Body composition**				
Waist circumference, centimeters	34	97.5 ± 15.5 (70-126)	97.2 ± 13.4 (74-125)	0.72
Hip circumference, centimeters	34	103.9 ± 10.4 (79-126)	103.6 ± 9.8 (81-126)	0.59
Waist-hip ratio	34	0.94 ± 0.1 (0.77-1.26)	0.94 ± 0.1 (0.78-1.08)	0.88
Body fat, %	32	28.1 ± 8.7 (9.7-47)	27.8 ± 8.6 (10.3-49.7)	0.27
**Lipid profiles**				
LDL cholesterol, mmol/L	27	2.38 ± 0.7 (1.3-3.8)	2.38 ± 0.7 (1.2-3.7)	0.48
HDL cholesterol, mmol/L	27	1.27 ± 0.3 (0.78-1.83)	1.31 ± 0.3 (0.91-2.0)	0.96
Total-HDL cholesterol ratio	27	3.84 ± 0.8 (2.1-5.6)	3.76 ± 1.0 (2.2-5.7)	0.60
Triglycerides	27	1.34 ± 0.8 (0.5-4.2)	1.37 ± 0.7 (0.4-3.2)	0.26
**Glucose and glucose control**				
Blood glucose, mmol/L	28	5.79 ± 1.1 (4.4-9.4)	5.55 ± 1.0 (4-8)	0.35
Glycated hemoglobin, %	28	5.88 ± 0.7 (4.8-8.2)	5.94 ± 0.7 (4.9-7.5)	0.71
**Metabolic syndrome**, n	26	10 (39%)	8 (31%)	0.18

Changes in study outcomes across 2 time points: pre-program (0 months) to post-program (6 months)

Values are n (%) or mean ± SD (range). Abbreviations: HDL = high-density lipoprotein; LDL = low-density lipoprotein.

## Discussion

The important finding from this study is that a CR model of exercise and risk factor management is feasible after stroke, providing that appropriate adaptations are made to accommodate for participants with mild to moderate disability. In addition, consistent with more targeted, stroke-specific aerobic training programs, adapted CR is effective in improving cardiorespiratory fitness.

Results from the current study support the adaptation of CR to include individuals with a range of mobility restrictions post-stroke. CR is a well-established program of care for individuals with cardiac conditions that is effective in reducing mortality [24,25], improving lipid profiles, BP [24] and fitness [26]. Stroke and heart disease share cardiovascular etiologies and risk factors, yet the availability of analogous secondary prevention programs are lacking in stroke care. Stroke survivors have little opportunity for fitness-based pursuits in traditional facilities [27] and CR programs that may include individuals with stroke, do so in a limited manner [5].

While baseline neurological indices characterized the study sample as mild to moderate in stroke severity, mobility assessments revealed that ambulatory capacity was considerably compromised. To accommodate the range of walking abilities among participants, adaptations in training modality and higher staffing ratios were employed. Utilizing alternatives for exercise testing and training (e.g. recumbent cycle ergometry) permitted individuals with limited ambulatory capacity to participate. Smaller class size and lower staff-to-participant ratio provided an adequate balance between close supervision, guidance, and encouragement to become more independent over time for the stroke participants. We observed low attrition and adverse event rates, and high levels of class attendance and participant satisfaction. Indeed the rate of program completion (93%) observed in the current study is higher than the 50-70% cited in the CR literature [28,29].

The adapted CR program, conducted in a community rehabilitation setting with a single on-site group format session, led to improvement in aerobic capacity over time. Pre-program fitness levels were below the metabolic energy requirements needed to perform many ADL's [30], but did exceed the 15 ml·kg^-1^·min^-1 ^threshold [31] by program completion. The mean change in VO_2_peak was lower than overall average change of 3.5 ml·kg^-1^·min^-1 ^reported in a recent Cochrane review that combined the results of randomized controlled exercise trials for individuals with stroke [32]. The magnitude of change was, however, comparable to other non-CR exercise interventions that may have used study methodologies other than randomized controlled trials that relied on multiple weekly on-site exercise sessions [13,33-35]. Macko and colleagues [35] demonstrated improvements in VO_2_peak and 6MWT distance with aerobic treadmill training but these sessions were not conducted in a group format. Group classes, such as an 8-week water-based exercise [36] and a 19-week fitness and mobility exercise program [37] increased post-stroke aerobic capacity by 23% [36] and 9% [37] and 6MWT performance by 65% [37] but, in both of these trials, interventions were comprised of 3 on-site exercise sessions per week and did not include any home-based component. The benefits of 10 weeks of supervised versus unsupervised exercise programs after stroke was examined in another study, and comparable effects were observed in both groups with respect to 6MWT performance (11% and 17% improvement in the supervised and unsupervised groups, respectively) and self-reported physical health [38]. While progressive aerobic exercise was a major component of this intervention, cardiorespiratory fitness was not explicitly measured [38]. Only one pilot study has specifically examined the use of a CR model after stroke, where the 10-week intervention of twice weekly on-site sessions and supervision at a 1:2 staff-to-participant ratio resulted in benefits to aerobic capacity and cardiac risk [39]. A full-scale trial has been proposed [40].

It should be noted that the change in VO_2_peak observed across the baseline period (change 1.2 ml·kg^-1^·min^-1^) was only slightly lower than the magnitude of pre- to post-program change (change 1.4 ml·kg^-1^·min^-1^), raising into question the true effectiveness of the intervention on aerobic capacity if there was improvement during the baseline observation period. Issues with measurement error or test-retest reliability of the maximal exercise tests in the stroke population may offer a partial explanation, but a wide range in intraclass correlations have been reported for repeated maximal exercise testing among individuals with stroke [41-43]. It is not known if a portion of the change in VO_2_peak observed during the baseline period in the current study may be attributed to a practice effect, as only 1 trial of the maximal exercise test was conducted. The high variation in individual participant responses in VO_2_peak across the 3 different time points may also explain why the changes observed across the baseline period were not statistically significant. Given this large variation, future study using a larger sample size may provide insight into the true effects of this adapted CR program.

There are important advantages to adapting the existing CR model to stroke. Most important is the opportunity to provide programs to these individuals using existing infrastructure and expertise in cardiovascular risk factor management through education and exercise [44]. In addition, CR has well-established approaches to maximize the 'at home' component of training that optimizes benefits but limits resource demands (versus a model of multiple weekly on-site training sessions). In the current study, we observed a successful combination of both on-site and at-home exercise sessions for a broad range of participants. Improvement in aerobic capacity is a key finding considering 80% of exercise sessions were unsupervised. Training intensity was lower than the intended target of 60-80% of HRR, although it has been suggested that lower intensity exercise (as low as 40% of HRR) may be sufficient to derive aerobic benefit among those with stroke [2,45].

The increase in aerobic capacity did not translate to improvements in functional ambulation as hypothesized. The variability of 6MWT performance may partially account for the lack of significant findings. However, more importantly, the CR program strove to improve cardiorespiratory capacity with no structured components to improve neuromotor control. Previous studies focused on post-stroke aerobic training have also revealed little benefit to neuromotor control [13,46]. Future research may delineate specific training that concurrently addresses cardiorespiratory fitness and walking ability after stroke.

Post-program changes in risk factors did not reach significance but, as noted, participants did demonstrate slight reductions in percent body fat and fasting plasma glucose. In addition, 2 participants no longer met the criteria for the metabolic syndrome by the end of the study. In an earlier study, improvements in indices of insulin sensitivity have been demonstrated with aerobic treadmill training after stroke but changes in fasting glucose were not observed [47]. Rimmer and colleagues [48] reported improvements in resting BP and total cholesterol particularly with moderate intensity short duration aerobic exercise after stroke; triglyceride levels were reduced with moderate intensity short duration and low intensity long duration exercise. Lennon et al [40] reported improvement in overall cardiac risk score after CR intervention with stroke, but not in the individual risk score components. The changes observed in the current study are encouraging given the small sample size; further study is warranted with a larger participant pool sufficiently powered to determine if a CR model may mediate risk for recurrent stroke.

There are several limitations to this study. As this study used a single cohort design, one limitation is the lack of control group. While not a considerable limiting issue for the purpose of establishing feasibility of the adapted CR program, future study to further establish effectiveness of this model of care would benefit from an independent control group. With regards to the exercise diaries, the conservative estimate used in assuming that "0" minutes of exercise was performed if a diary was not submitted may have underestimated the actual amount completed. As well, the variance in 6MWT scores was unexpectedly higher than what was used for calculation of sample size. Thus, we were likely underpowered to detect changes in this outcome, in addition to changes in stroke risk factors as previously mentioned. Further, changes in muscular strength and endurance and thus the role of resistance training on study outcomes are not described here. Finally, analysis of follow-up data to determine if the observed benefits were retained after the program ended is also warranted.

### Implications

There is currently a dearth of opportunities for stroke survivors to receive guidance on safe and effective exercise to lower recurrent stroke risk. CR may be an innovative opportunity to translate a successful and well-established model of care across both sides of cardiovascular disease management. The applicability of CR to stroke stems from the commonalities between stroke and heart disease. Given the cardiovascular etiology of stroke and presence of shared co-morbidities and risk factors, a CR model of care may fill this gap in stroke care. This study is an important first step in designing effective long-term community-based secondary stroke prevention programs. We demonstrated that CR may be feasibly adapted to accommodate for individuals with a wide range of functional abilities post-stroke and is effective in increasing aerobic capacity.

## Conclusions

In summary, a CR model of care may offer community-based exercise and education programming for risk factor management and can be adapted to accommodate individuals with mild to moderate stroke disability. Despite using only once weekly, supervised exercise sessions supplemented with 4 sessions of independent at-home exercise per week, this adapted program is effective in increasing aerobic capacity. CR may be an untapped opportunity for stroke survivors to access programs of exercise and risk factor management.

## Competing interests

The authors declare that they have no competing interests.

## Authors' contributions

AT, as primary author, participated in study design, was responsible for writing the manuscript, data collection, analysis and interpretation. SM participated in data collection and analysis and interpretation. PO participated in study design, assisted with medical screening and data collection and interpretation. WEM conceived of the study, assisted with data analysis and interpretation. DB conceived of the study, assisted with data analysis and helped draft the manuscript. All authors have read and approved the final manuscript.

## Pre-publication history

The pre-publication history for this paper can be accessed here:

http://www.biomedcentral.com/1471-2377/10/40/prepub
